# Parasitic Zoonoses in Humans and Their Dogs from a Rural Community of Tropical Mexico

**DOI:** 10.1155/2015/481086

**Published:** 2015-12-07

**Authors:** Antonio Ortega-Pacheco, Juan F. J. Torres-Acosta, Alejandro Alzina-López, Eduardo Gutiérrez-Blanco, Manuel E. Bolio-González, Armando J. Aguilar-Caballero, Roger I. Rodríguez-Vivas, Edwin Gutiérrez-Ruiz, Karla Y. Acosta-Viana, Eugenia Guzmán-Marín, Alberto Rosado-Aguilar, Matilde Jiménez-Coello

**Affiliations:** ^1^Departamento Salud Animal y Medicina Preventiva, Cuerpo Académico en Salud Animal, Facultad de Medicina Veterinaria y Zootecnia, Universidad Autónoma de Yucatán, Km 15.5 Carr. Mérida-Xmatkuil, AP 4-116, Mérida, YUC, Mexico; ^2^Laboratorio de Biología Celular, CA Biomedicina de Enfermedades Infecciosas y Parasitarias. C.I.R. “Dr. Hideyo Noguchi”, Universidad Autónoma de Yucatán, Avenida Itzaes, No. 490 x C. 59, 97000 Mérida, YUC, Mexico

## Abstract

A cross-sectional study was made on 89 inhabitants and their dogs from a rural community of Yucatan, Mexico, to determine the serological prevalence of some zoonotic parasitic agents. Samples were taken to monitor the presence and intensity of infection with gastrointestinal parasites in dogs. In humans, the serological prevalence of* T. canis*,* T. gondii*, and* T. spiralis* was 29.2%, 91.0%, and 6.7%, respectively. No associations were found between positive cases and studied variables. From the total of blood samples taken from dogs, 87 (97.6%) were seropositive to* T. gondii*; only 52 viable fecal samples were collected from dogs of which 46.2% had the presence of gastrointestinal parasites with low to moderate intensity; from those, 12% had the presence of* T. canis*. This study demonstrates the presence of the studied zoonotic agents in the area particularly* T. gondii* which suggest a common source of infection in dogs and humans and a high number of oocyts present in the environment. Preventive measures must be designed towards good prophylactic practices in domestic and backyard animals (*T. canis* and* T. spiralis*). Contaminated sources with* T. gondii* (food and water) should be further investigated in order to design effective control measures.

## 1. Introduction

Dogs are the source of several zoonotic diseases and their close contact with humans may increase the risk of zoonotic disease transmission particularly when dogs are free to roam. Dogs in urban and rural areas can be used as sentinels in humans and wildlife to evaluate the diversity and environmental contamination with parasites [1]. Backyard pigs in tropical Mexico are important for domestic consumption [2, 3]. However, their sanitary management is very poor or nonexisting [3, 4] representing a risk for the transmission of zoonotic parasitic agents. The parasites* Toxoplasma gondii* and* Toxocara* spp. are capable of producing systemic and ocular disease in dogs and humans and share soil ingestion as common mode of exposure [5]. However, the protozoa* T. gondii* are more commonly transmitted by the ingestion of contaminated food or water. Domestic dogs are the principal animal reservoir hosts for* Toxocara canis*, a common nematode throughout the world producing severe anemia and systemic disease in puppies which in humans elicits various syndromes currently characterized as generalized (visceral larva migrans and covert toxocariasis) and compartmentalized as well as ocular and neurologic [6], with more clinical manifestations on kids [7]. Diverse epidemiological studies have demonstrated that exposition to* T. canis* varies from countries and regions [8] reaching up to 83% of seropositivity in people from the Caribbean region [9]. Another important foodborne nematode* Trichinella spiralis* is transmitted to humans generally by the consumption of undercook contaminated pork meat [10].

Information related to both animal and human zoonotic parasite infections in rural communities of Mexico is scarce. To establish an effective parasite prevention and control strategies, it is necessary to understand the biology of parasites circulating in endemic regions, monitoring their presence and the risk factors associated in humans, reservoir animals, and vectors for a better planning of strategies for prevention and control of zoonotic parasitic diseases. The objective of this study was to determine the seroprevalence of humans to* T. canis*,* T. gondii*, and* T. spiralis* in a rural community of Yucatan, Mexico, together with the seroprevalence of* T. gondii* and detection of* T. canis* in their dogs.

## 2. Methods

### 2.1. Area of Study

The study was conducted in the rural community of Molas Yucatan, Mexico (20°49′ and 20°48′N and 89°37′ and 89°38′W). The community has 2014 inhabitants and 379 dwellings. A sectional, descriptive, and prospective study was conducted considering for convenience 90 dwellings reporting tenure of at least one dog. The community was divided into four sectors (north-south and east-west); 20 houses in each sector were selected.

### 2.2. Sampling

Each of the selected households was visited, and a directed questionnaire was applied. Samples of blood and feces of at least one dog per household were taken. In dogs, blood samples were obtained from the cephalic vein and were processed to obtain serum which was frozen at −20°C until tested. Feces were taken directly from the rectum; then, rabies vaccine was applied and dogs were dewormed. Additionally, blood samples from an adult person per household were taken. In humans, blood samples were collected by venipuncture of the brachial vein prior to written consent of the participants and according to the rules of the Bioethics Committee of the Campus of Biological and Agricultural Sciences at the Autonomous University of Yucatan (CB-CCBA-I-20014-001). Samples were processed to obtain serum and frozen at −20°C. A questionnaire was applied to all sampled persons including questions about their age, feeding habits, water sources, presence of eye lesions, and previous deworming procedures.

### 2.3. Laboratory Analysis

Fecal samples were processed by centrifugation-flotation technique [11]; positive samples were examined quantitatively by McMaster technique to estimate the number of eggs per gram of feces (epg) with particular attention to* T. canis*. Human sera were evaluated for the measurement of IgG* T. canis* and* T. spiralis* antigens using ELISA kit (DGR Laboratories International Inc., USA) following the manufacturer's instructions. For* T. gondii*, the presence of specific IgG antibodies was determined by indirect ELISA (International GmbH RE58241, Hamburg, Germany) containing tachyzoites of* T. gondii*. Serum samples were diluted to a ratio of 1 : 100 in PBS and secondary antibody anti-human IgG HRP-labeled anti-human IgM HRP were also marked.

### 2.4. Statistical Analysis

The overall prevalence of intestinal parasites in dogs was estimated. The intensity of infection (elimination of epg) was classified as low (50–100 epg), median (150–500 epg), or high (>550 epg). The overall prevalence of IgG-positive people to* T. canis*,* T. canis*, and* T. spiralis* was also estimated. A primary screening of humans seropositive to* T. canis* was assessed using 2 × 2 contingency tables of exposure variables; odds ratio and confidence interval were determined. Since very few negative cases of* T. gondii* in human and dogs and few positive cases of* T. spiralis* were found, no statistical analysis was performed. Odds ratios (OR) and 95% confidence intervals were calculated using Epi-Info software (version 6.0; CDC Atlanta, GA, USA). The level of significance was set at *P* < 0.05.

## 3. Results

A total of 52 viable fecal samples were collected from adult dogs of which 24 (46.2%) had at least some order/species kind of intestinal nematode. The nematode genera found in dog feces were* Ancylostoma caninum* (70.8%),* Toxocara canis* (12.5%),* Trichuris vulpis* (12.5%), and the order Eucoccidiorida (4.2%).

The intensity of infection of nematode order/species is presented in Table 1. In most cases, intensity of the infection was low followed by median intensity; no cases of high infection load were seen; from the infected dogs, all of them had only a single order/species of nematode present.

Regarding the serology from human samples, 26 (29.2%) from the 89 persons were positive to IgG anti-*T. canis* (Table 2). None of the risk factors studied were significantly associated with seropositivity in humans (Table 3). All participants owned at least one dog and none of them collected the feces from their animals. From the questionnaire, 36 persons claim vision disorders such as cloudy vision and increased tearing.

Regarding IgG anti-*T. gondii* serological results, from 89 human blood samples collected, 90.6% were positive to IgG* T. gondii* (Table 2). For dogs, 89 samples were obtained and 97.6% were seropositive to* T. gondii*. Since very few negative cases were found, no statistical inferences were feasible. From the interviews, 93% drink water from commercial carafes, 5% drink tap water, and 2% drink from both sources. Participants reported that the ingestion of meat is usually well cooked in 82% of cases and 7% claim to eat semicooked meat. 88.8% of the interviewed persons eat sausages and from the 10 persons (11.2%) who do not eat sausages 2 were seronegative.

Only 6 (6.9%) persons were found seropositive to* T. spiralis* (Table 2).

## 4. Discussion

The prevalence of dogs infected with gastrointestinal parasites in this study is considered high and similar to the reported one in other regions of the world, that is, 52.4% in Argentina [12], 53% in Hungary [13], and 68% in Nigeria [14]. The high variation observed has been associated with various weather conditions, idiosyncrasies, and sanitary control of dogs. A previous study in the same community was performed four years ago and prevalence of gastrointestinal parasites in dogs was 80% with a high egg output of* A. caninum*,* T. canis*, and* T. vulpis* found; in this study, deworming of dogs was performed and talks to children, adolescents, and adults about health management of dogs were given [15]. These actions could explain the lower prevalence and intensity of infection reported at this time categorized between low and medium intensity of infection. In the specific case of* T. canis*, a higher prevalence (12.5%) was found compared to previous studies in the same area where 6.2% was found [15].* T. canis* mainly affects puppies with frequently fatal consequences. In adult dogs, the infection is minor and usually asymptomatic. The annual variation in the number of infected puppies in the region may influence the disposal and spreading of* T. canis* eggs. The presence of infected puppies can increase the number of eggs and larvae deposited in the environment in which they can remain infective for several months infecting other dogs and with great potential to infect humans, especially children.

Human toxocariasis is worldwide distributed and occurs when embryonated eggs from the environment are ingested. This is more critical when dog overpopulation is present without the practice of preventive medicine (deworming). Children with pica are found at increased risk particularly in regions of low socioeconomic status and with proliferation of dogs and/or those who have puppies at home [7]. Adults are also at risk of infection as shown by several studies where prevalence of 26.8% in Brazil was reported [16] and 19% in Lebanon was reported [17]. In Argentina, seroprevalence of 20–38% in children and 10–39% in general population [18, 19] is reported. In Mexico, 38% of people suffering from acute nongranulomatous uveitis were seropositive for* T. canis* [20], with a prevalence of 4.6% to 17.59% reported in children less than 16 years of age [21]. Results of this study indicate a high spread of infective eggs in the rural community probably because of the high density of dogs having free access to the street and lack of health programs including a deworming program.

Although deworming has been introduced in health management of dogs in the studied community, still spreading of infected eggs and contamination of the area are present; thus, efforts to intensify the deworming of dog and cat are necessary for better control results. The ocular larva migrans syndrome is one of the major health problems associated with* T. canis* [22]. However, eye lesions were not statistically associated with seropositive persons; this syndrome is more critical in children where severe problems have been reported [7], so studies to evaluate the impact of this parasite on children should be designed and performed.* T. canis* and* T. gondii* are associated with eye lesions such as uveitis [23]. Ocular larva migrans in the case of toxocariasis may cause visual impairment as a response of granulomatous inflammatory reaction with consequences such as blindness and secondary glaucoma [7, 24]. In the present study, no association was found in patients seropositive to* T. canis* and vision disorders. However, other symptoms not evaluated in the present study may involve headache, muscle pain, influenza-like syndrome, and diarrhea in seropositive patients [25]. In a previous study conducted in Mexico,* T. canis* positive patients were highly associated with ankylosing spondylitis [26]. Young children, <5 years, are most affected with toxocariasis with clinical signs of fever, abdominal pain (probably due to hepatomegaly), and lower respiratory symptoms [7]. Further studies should be focused on children in the studied region examining the different body systems to evaluate the impact of this zoonotic agent.

The high prevalence of* T. gondii* found in dogs and their owners demonstrates a high spread of oocysts in the studied area with a probably common source of infection. The prevalence found in dogs from Brazil was 26.9% with a higher risk for dogs over five years of age [27]. However, in the present study, due to the very high prevalence found, all animals are expected to seroconvert since young ages with frequent episodes of reinfection. In Mexico, dog from shelters in Veracruz also showed a high seroprevalence reaching up to 67.3% [28]. Free roaming dogs are indicators of the high contamination of the environment with* T. gondii* oocysts as they may be sentinels of exposition. In humans, toxoplasmosis has been reported previously in the Yucatán peninsula with seroprevalence of 25% in non-cat owners [29] and also associated in patients with recent cases of spontaneous abortion [30, 31]. Seroprevalence may reach up to 87.3% in humans from rural communities from Yucatan [32]. In humans, the main route of infection of* T. gondii* is by the ingestion of tissue cysts [33]. In the present study, the oral route by consumption of undercooked meat (tissue cysts) is more likely but also the ingestion of infective oocysts from contaminated water as previously demonstrated could occur [34]. Considering the 100%* T. gondii* seroprevalence found in 50 studied cats from the same region [4], the density of cats and constant new born kittens in the village with excretion of high numbers of oocysts probably occurred, contaminating the environment and probable water sources. Even when 93% of the interviewed persons drank commercial bottled water, tap water and water from artisanal wells are also used for several other activities and may be considered as a potential source of infection with* T. gondii* oocysts. However, since dogs in the studied area are fed with human leftovers [35], contaminated meat and sausages may also be involved in the transmission cycle.

*T. spiralis* is a nematode capable of infecting any mammal ingesting infected raw or undercooked meat. Infection in humans with this nematode is described worldwide and outbreaks have been reported in developed countries infecting over 30% of people ingesting wild boar meat [36]. Exposition of consumers to infected meat can reach up to 65.1% of incidence, especially when meat is not tested prior to consumption when slaughter is made in households [37]. In Mexico, consumption of pork meat coming from backyard is very common in rural villages without any kind of quality test [4]. In Mexico, the Ministry of Health (NOM-194-SSA1-2014) makes the inspection of pork meat in search of* T. spiralis* compulsory. However, this is rarely done at the abattoirs or before commercialization.* T. spiralis* in Mexico has been reported since 1975 in Zacatecas state where several outbreaks have been reported in cases with mortality rates of 33% in some cases; in those outbreaks, transmission was most commonly caused by the ingestion of raw pork products or undercooked meat [38]. More recently, a prevalence of 1% using ELISA was reported in semirural county areas of Mexico [39]. In Yucatan, no proper surveys of* T. spiralis* have been performed in humans or animals. This is the first report of* T. spiralis* serological presence in humans from Yucatan. However, the* Toxocara* larval excretory antigen is normally used in serological tests that may exhibit low specificity due to cross-reaction between related helminthes, including* T. canis* [40]. Further research is needed to investigate the epidemiology of these zoonotic helminthes using more accurate techniques for diagnosis including the search of the agent in farm and backyard pigs from the region.

The main limitation of the present study was the small sample size used and consequently results may be biased or may not be representative of the rural communities from the studied area. However, this study may be a good indicator of the zoonotic situation and establish the bases for further studies including a bigger and targeted sample size from both animals and humans.

## 5. Conclusion

It is concluded that the dogs in a rural community of Yucatan are reservoirs of some helminthes parasites specially* T. canis*. This implies a high risk of the presence of parasites in the environment of the village and a potential risk of infection for the human community. It is important to improve the dissemination and promotion of educational programs on this important zoonosis and establish effective control measures to reduce the emission of eggs and avoid contact of human with areas contaminated with dog feces. Other important zoonotic parasites (*T. gondii* and* T. spiralis*) are also circulating.

## Figures and Tables

**Table 1:** Nematode infection intensity (epg) and Eucoccidiorida (oocysts per gram of feces) in 52 dogs from a rural community of Yucatan, Mexico.

Intestinal parasites	Low	Median	High
*Ancylostoma caninum*	45.8%	25.0%	0.0%
*Toxocara canis *	12.5%	0.0%	0.0%
*Trichuris vulpis*	8.3%	4.2%	0.0%
Eucoccidiorida	0.0%	4.2%	0.0%
Total	66.6%	33.4%	0.0%

Low 50–100 e/g, median 101–500 e/g, and high ≥500 e/g.

**Table 2:** Serological frequency of zoonotic agents identified in 89 persons from a rural community of Yucatan, Mexico.

Agent	Positive cases (*n*)	%
*T. canis*	26	29.2
*T. gondii*	81	91.0
*T. spiralis*	6	6.7
*T. canis* + *T. gondii*	26	29.2
*T. spiralis* + *T. gondii*	4	4.5

**Table 3:** Relationship between associated factors with *T. canis* IgG seropositivity in humans from a rural community of Yucatan, Mexico.

	*n*	Positive	Prevalence	OR	95% CI	*P* value
Age (years)						
20–40	29	8	27.6%	1.5	0.54–4.26	0.42 (NS)
>40–75	55	11	20.0%	—	—	—
Sex						
Male	8	1	12.5%	—	—	—
Female	76	18	23.7%	4.46	0.05–3.9	0.41 (NS)
Eyes lesions						
Yes	52	16	30.8%	1.3	0.49–3.6	0.56 (NS)
No	32	8	25.5%	—	—	—
Deworming						
Yes	32	12	37.5%	1.8	0.91–6.02	0.22 (NS)
No	52	13	25.6%	—	—	—

*n*: number of humans studied, OR: odds ratio, CI: confidence interval, and *P*: probability.

NS: not significant.
